# Strategies and effects of promising school-based interventions to promote active school transportation by bicycle among children and adolescents: protocol for a systematic review

**DOI:** 10.1186/s13643-019-1216-0

**Published:** 2019-11-29

**Authors:** Dorothea M. I. Schönbach, Teatske M. Altenburg, Mai J. M. Chinapaw, Adilson Marques, Yolanda Demetriou

**Affiliations:** 1 Department of Sport and Health Sciences, Technical University of Munich, Georg-Brauchle-Ring 62, D-80992 Munich, Germany; 2 Amsterdam UMC, Vrije Universiteit Amsterdam, Department of Public and Occupational Health, Amsterdam Public Health research institute, Amsterdam, The Netherlands; 3 Interdisciplinary Centre for the Study of Human Performance, Faculty of Human Kinetics, University of Lisbon, Lisbon, Portugal

**Keywords:** PRISMA-P, Program, Educational facilities, Pupil, Active school travel, Biking, (Randomized) controlled trial

## Abstract

**Background:**

Active school travel by bike may provide appropriate means to promote physical activity through commuting to and from school, expanding the mobility during leisure time, and integrating a lifelong positive behavior routine. However, bicycling seems to be a less common form of active school transport and declining cycling to school trends in some European countries have been observed. Therefore, effective interventions aiming at promoting biking to school are warranted. To gain a better understanding of effective programs, the systematic review will summarize strategies and effects of school-based interventions targeted on positively influencing active school travel by bicycle.

**Methods:**

The databases ERIC, PsycINFO, PSYNDEX, PubMed, Scopus, SPORTDiscus, SURF, and Web of Science will be searched utilizing a detailed search strategy according to “PICo”. Consequently, there will be no restriction regarding the outcomes measured in studies. For inclusion in the review, the identified primary studies (i.e. randomized and non-randomized controlled trials) should be published between 2000 and 2019 due to their current relevance, and written in English. The screening, data extraction, and appraisal of study quality as well as behavior change techniques will be undertaken by two independent researchers. To assess the methodological quality of every included study, the quality assessment tool “Effective Public Health Practice Project” for quantitative studies will be used. Behavior change techniques will be identified by utilizing the “BCT Taxonomy v1”. If data permits, meta-analyses for intervention effects will be conducted where appropriate.

**Discussion:**

The planned systematic review can provide information about how bicycling is considered in school-based interventions as an effective strategy to promote active commuting to school among students. In this regard, the conclusions drawn from the review will establish a basis for researchers to plan and implement a comprehensive cycling intervention in the school setting.

**Systematic review registration:**

PROSPEROCRD42019125192

## Background

Despite the well-proven health benefits of physical activity (PA) in childhood and adolescence [1], most young people in Europe still do not meet the PA recommendation of the World Health Organization [2], which includes 60 min of moderate-to-vigorous intensity PA accumulated every day [3]. The low compliance with the PA recommendation is alarming as all health benefits of PA appear to have their origin in early life [4] just as various health problems in adulthood, like overweight or obesity [5]. Given that the development of active habits in this period of life is expected to remain stable [6–10], the effort of PA promotion has to occur as early and continuously as possible.

Schools are an ideal setting for promoting PA [11–14]: Firstly, school attendance is compulsory in many countries [15]. As a result, all children and adolescents can be reached regardless of their social background and they have to commute to and from school each day [16]. Secondly, students spend about half of their day at school since the implementation of full-time schools in most countries [12] and therewith, they may have less time to engage in leisure time PA according to the displacement hypothesis [17]. Due to the educational mandate at schools, the Institute of Medicine published an international PA recommendation for schools according to which traveling to and from school is highly recommended for pupils as an additional opportunity for being physically active [18].

Active school travel (AST) could be a meaningful long-term possibility to promote PA [18]. Current research has indicated that AST is positively associated with PA levels per day [19–22], per school day [20, 23], and immediately before and after school [20, 21, 23]. Especially female adolescents seem to benefit from engaging in AST [20]. Additionally, a potentially lifelong habit of active transport in general may be established as a result of a daily AST routine in early years [16]. Furthermore, AST provides favorable health benefits, such as a positive effect on body composition [21, 24]. Simultaneously, AST may also have a positive impact on reducing traffic [25, 26], which consequently protects the environment concerning air pollution [16, 26] and increases road safety [25]. But since the development of intervention studies in this area of research is still in an early stage [27], the promotion of AST has been described as the least implemented measure up to now, especially in secondary schools [28]. This circumstance may explain the current lack of knowledge about the effectiveness of intervention studies in the long term [27] despite cross-sectional findings of increased AST rates when schools supported AST behavior [29].

One option of AST besides walking is cycling. Lately, it was reported that bicycling makes a positive contribution to improving cardiovascular fitness in children and adolescents aged 5 to 17.9 years due to its higher intensity compared with walking [16] as well as to meeting the international PA recommendation [22]. Thirty-six percent of AST cyclists aged 5 to 15 years achieved the guideline per week with a mean weekly cycling-related AST time of 1.4 h, which contributes 20% to the recommended weekly minutes [22]. Thus, cycling-related AST may be promising for decreasing future risk of cardiovascular diseases. Regarding bicycle ownership in this context, between 57 and 98% of children and adolescents aged 0 to 17 years already own a bicycle in Germany for instance [30]. Accordingly, bicycling seems to be a cost-effective form for students to get to and from school [16]. By contrast, the percentage of German students who actually use their bikes to cycle to school varies from 8 [31] to 22.2% [32] depending on the region with observed gender differences of 23.8% in boys versus 20.6% in girls [32]. Compared with cycling to school trends between 2006, 2010, and 2014 among Czech schoolboys (5.7%, 3.2%, 2.2%) and schoolgirls (2.3%, 0.5%, 2%) aged 11 to 15 years [33], biking tradition seems to vary enormously between individual European countries. In accordance with the current state of research, the need for action with respect to cycling as a less common form of AST is warranted [33] and the negative development has also to be reversed.

Against this background, evidence-based interventions aiming at promoting biking to school are needed. To the authors’ knowledge, none of the previously published systematic reviews dealt exclusively with cycling as a mode of AST, whereas there is already one that focused on walking in particular [25]. Hence, this review will summarize strategies and effects of school-based interventions to promote AST by bicycle among children and adolescents, which follow a pretest-posttest comparison group design.

## Methods

This systematic review protocol has been registered in the international prospective register of systematic reviews called PROSPERO (registration number: CRD42019125192). For the preparation of the protocol, the checklist “Preferred Reporting Items for Systematic review and Meta-Analysis for Protocols” [34] was utilized (PRISMA-P: see Additional file 1). Any discrepancies in the announced procedure of this protocol will be documented and published within the final review and PROSPERO.

### Search strategy

The search strategy will be designed in collaboration with two specialists employed at the information services in the University Library (Technical University of Munich) and will be based on “PICo” [35]. According to the three factors of “PICo”, three groups of search terms will be defined that have to be integrated in title or abstract. The combinations of keywords related to population, interest, and context are illustrated in Additional file 2. To identify potentially relevant primary studies, the systematic literature search will be conducted in the following eight electronic databases: ERIC, PsycINFO, PSYNDEX, PubMed, Scopus, SPORTDiscus, SURF, and Web of Science. All search results given by the utilized electronic information sources will be restricted to English language and will be limited to studies published between 2000 and 2019 due to their current relevance.

### Eligibility criteria

Only studies will be included whose school-based intervention components pursue the goal to increase the use of bicycles during the school travel as appropriate means of promoting AST, such as an adult-guided cycling route to and from school. In this context, the term “school-based” is defined as everything that happens in or on the way to and from school but school staff (e.g. teachers) do not necessarily have to be involved. Intervention outcomes can be quantified by any type of common measures (e.g. questionnaires, accelerometers, interviews, tests, cycle computer) and will not be restricted to a predefined issue. In addition, only samples targeted on primary and/or secondary schools will be taken into consideration. Moreover, randomized controlled trials (RCTs), in terms of parallel-group or cluster-randomized, and controlled trials (CTs) that represent children and adolescents without specific health issues will be included in the systematic review. The comparators should be either an active control group, for example receiving an intervention to promote young people’s creativity or cognitive performance without components promoting AST, PA or reducing sedentary behavior, or a control group with no intervention. Finally, all of the included studies must have provided intervention effects analysis by comparing pretest and posttest values between intervention and control group.

### Study selection

All identified records will be imported into EndNote, and duplicates will be removed. Then, the identified references will be screened by two independent reviewers (DS and TA) in consideration of the described eligibility criteria for inclusion following three steps based on title, abstract, and full text. If necessary, any potential discrepancies during these three steps of the selection process will be resolved by discussions between DS and TA after re-examination of studies, or in case of continued disagreement by a third independent reviewer (YD). Authors will be contacted a maximum (max.) of two times via e-mail when articles are not available, or relevant details are missing in the article.

### Data extraction

Specific study details for each included full text pertaining the two research questions will be listed in a spreadsheet (excel) by DS and TA/AM. Prior to this, the spreadsheet will be piloted on the basis of three randomly selected full texts to ensure consistency among the two independent data extractors as well as to ensure a systematic process during data extraction. Information will be entered into the table, such as general study details (i.e. author, country, year, design, study aim), theoretical background, characteristics of participants (i.e. total/subgroup sample size/s, sample size determination, class level/age, stage of life, participantsʼ recruitment/retention rate), intervention description (i.e. name, components, approach, behavior change technique (BCT), tasks of control group, duration, frequency, points of data collection), statistical analysis (incl. confounder), and measuring instruments as well as effects of individual intervention outcomes. With reference to BCT, two independent evaluators (DS and TA) will code intervention strategies applied in all included studies utilizing the “BCT Taxonomy v1” [36], which consists of 93 hierarchically clustered techniques. Any discrepancies between both evaluators will be resolved by discussions, or if needed, by consulting a third independent evaluator (YD). While extracting the data, the data extractors will not be blinded to authors and journals.

### Quality assessment

The component-based quality assessment tool “Effective Public Health Practice Project” [37] for quantitative studies will be used for assessing methodological quality of all included primary studies.

Critical judgements will be made separately for all items within the eight sections/components shown in Additional file 3. DS and TA/AM will rate the methodological quality for each item as strong, moderate or weak according to standardized instructions published in the associated tool dictionary. Any discrepancies between the evaluators (DS and TA/AM) regarding the individual rating of items will be resolved through discussion. As the final review will only include RCTs or CTs, the item “study design” will be rated as strong for all included studies and will only be used to separate RCTs from CTs. With reference to the eight selected confounders based on the “Model of Childrenʼs Active Travel” [38] mentioned in Additional file 3, this item will be assessed as strong when at least five relevant confounders are considered in the study, moderate when between three and four relevant confounders are taken into account, and weak when less than two relevant confounders are reported. Based on the current evidence of controlled confounders, the quality of this item will be rated as strong for gender [33] and migration background [32] whereas age [33, 39] and previous AST experiences at baseline level [40, 41] will be rated as moderate, and all other variations will be rated as weak.

After rating all individual items, each of the eight quality components will be assessed as strong (more strong than moderate ratings and no weak ratings), moderate (not more than one weak rating), or weak (two weak ratings or more). The global quality of a study will be rated as strong when at least five components are assessed as strong and no components are assessed as weak [42]. When less than five components are assessed as strong and one component is assessed as weak, the global quality of a study will be rated as moderate [42]. The methodological quality of a study will be rated as weak when two or more components are assessed as weak [42]. Even though the studies will be assessed according to their quality, they will not be weighted [43]. As a result, findings from studies with weak quality will not be given less importance than findings from studies with strong quality [43].

### Data synthesis

The included articles in the systematic review will focus on various intervention characteristics and outcome variables collected by utilizing diverse measuring instruments. This is why conducting a narrative synthesis to describe and summarize the findings of these studies is expected to be the most appropriate method. The following two criteria will be used to decide whether or not a meta-analysis will be integrated: (a) Sufficient content-related homogeneity regarding similar research questions is necessary among studies. (b) To reach a good approximation in terms of statistical distributions, the minimum (min.) number of studies is set at five. In case of performing a meta-analysis, the software “R” with its packages “meta(for)” will be used. Study details, methodological quality assessments (separated into sectional and global rating), and intervention approaches (behavior change strategies) of the relevant studies will be illustrated in tables. Furthermore, it is planned to group the various outcome variables (e.g. AST behavior, overall PA levels, physical fitness, accident rates, knowledge about bike-specific traffic rules, bike-specific motor skills). A separate analysis for children and adolescents will also be performed. According to the study by Van Hecke et al. (2016), children will be defined up to 12 years of age and adolescents from 13 years of age [2]. Due to different school ages in different countries, we will not restrict the age group to a range with a min. or max. value. If reported in studies, we will consider gender and regional differences as well. This procedure is aimed at gaining insights into potential age, gender, or cultural dynamics in intervention strategies that have already been implemented and were successful in increasing AST.

## Discussion

The planned systematic review will critically evaluate the literature on school-based bicycle intervention strategies and their effects on a variety of outcomes, such as AST behavior, overall PA levels, physical fitness, accident rates, knowledge about bike-specific traffic rules, or bike-specific motor skills. An extensive overview of existing studies on promising school-based bicycle intervention strategies and their effects is required for increasing the prevalence of bicycling as an important form of AST among children and adolescents.

We anticipate that the planned systematic review will have some limitations at study as well as review level. At study level, potential limitations could include intervention strategies that are not based on a theoretical framework or described in detail, heterogeneity in applied strategies or outcomes, small number of long-term studies, small sample sizes that limit representativeness, and low study quality. We further expect potential limitations at review level, like small total number of studies, inappropriateness of meta-analyses due to a variety of statistical units and analyses, or weak evidence for effectiveness. Nevertheless, the systematic review can make a contribution to closing an existing research gap.

The findings of the review will be disseminated through the publication in an international peer-reviewed journal, formal presentations at conferences, and informal meetings. In addition, the findings of the review will be used to make recommendations that will immediately be transferred into an evidence-based best practice example of a school-related AST intervention in our European project.

## Supplementary information

**Additional file 1.** Preferred Reporting Items for Systematic review and Meta-Analysis for Protocols (PRISMA-P) 2015 checklist: recommended items to address in a systematic review protocol.

**Additional file 2.** Draft of the search strategy utilized in each selected database.

**Additional file 3.** Sections, components and items of the quality assessment tool.

## Data Availability

Not applicable

## Abbreviations

AST: Active school travel
BCT: Behavior change technique
CTs: Controlled trials
e.g.: Exempli gratia (for example)
i.e.: Id est (that is)
incl.: Inclusive
max.: Maximum
min.: Minimum
min: minutes
PA: Physical activity
PICo: Population, interest, context
PRISMA-P: Preferred Reporting Items for Systematic review and Meta-Analysis for Protocols
RCTs: Randomized controlled trials
