# Supplementary material for: Strategies and effects of promising school-based interventions to promote active school transportation by bicycle among children and adolescents: protocol for a systematic review
Source: Syst Rev. 2019 Nov 29;8:296. doi: 10.1186/s13643-019-1216-0 (PMC6884831; doi:10.1186/s13643-019-1216-0)
Supplement: Supplementary file 2 — Additional file 2. Draft of the search strategy utilized in each selected database. [file 13643_2019_1216_MOESM2_ESM.docx]

Additional file 2. Draft of the search strategy utilized in each selected database.

| **Search Strategy** | |
| --- | --- |
| **Population** | child* *OR* infan* *OR* adolescen* *OR* preadolescent *OR* juven* *OR* teen* *OR* young* *OR* youth* *OR* student* *OR* pupil* *OR* boy* *OR* girl* *OR* kid* *OR* pediatric* |
| *AND* | |
| **Interest** | intervention* *OR* training* *OR* experiment* *OR* program* *OR* education* *OR* treatment* *OR* evaluat* *OR* course* *OR* outcome* *OR* effect* *OR* result* *OR* evidence *OR* finding* *OR* impact* *OR* encourag* *OR* improv* *OR* increas* *OR* promot* *OR* enhanc*  *AND*  travel* *OR* transport* *OR* commut*  *AND*  cycl* *OR* bicycl* *OR* bik* |
| *AND* | |
| **Context** | school* *OR* class* *OR* physical education *OR* lesson* |
