# Supplementary material for: Strategies and effects of promising school-based interventions to promote active school transportation by bicycle among children and adolescents: protocol for a systematic review
Source: Syst Rev. 2019 Nov 29;8:296. doi: 10.1186/s13643-019-1216-0 (PMC6884831; doi:10.1186/s13643-019-1216-0)
Supplement: Supplementary file 3 — Additional file 3. Sections, components and items of the quality assessment tool. [file 13643_2019_1216_MOESM3_ESM.docx]

Additional file 3. Sections, components and items of the quality assessment tool.

| **Section** | **Component** | **Items** |
| --- | --- | --- |
| A | Selection Bias | representativeness of sample; percentage of recruitment rate |
| B | Study Design | RCT or CT; randomization; method of randomization; appropriateness of randomization method |
| C | Confounders | group differences prior to intervention; relevant confounders according to the “Model of Childrenʼs Active Travel” (38): age, sex/gender, previous AST experiences, weight status, migration background, bicycle ownership, socio-economic status, distance from home to school; quality rating of controlled confounders |
| D | Blinding | blinding of outcome assessor(s); blinding of participants |
| E | Data Collection Methods | validity of data collection tools; reliability of data collection tools |
| F | Withdrawals/ Drop-Outs | report of drop-outs (numbers/reasons); percentage of retention rate |
| G | Intervention Integrity | percentage of intervention delivery; measurement of interventionʼs consistency; contamination/co-intervention |
| H | Analyses | unit of allocation; unit of analysis; appropriateness of statistical methods; intention to treat |

RCT= randomized controlled trial; CT= controlled trial; AST= active school travel
